# West Nile virus infectious replicon particles generated using a packaging-restricted cell line is a safe reporter system

**DOI:** 10.1038/s41598-017-03670-4

**Published:** 2017-06-12

**Authors:** Wei Li, Le Ma, Li-Ping Guo, Xiao-Lei Wang, Jing-Wei Zhang, Zhi-Gao Bu, Rong-Hong Hua

**Affiliations:** State Key Laboratory of Veterinary Biotechnology, Harbin Veterinary Research Institute of Chinese Academy of Agricultural Sciences, Harbin, 150001 China

## Abstract

West Nile virus (WNV) is a neurotropic pathogen which causes zoonotic disease in humans. Recently, there have been an increasing number of infected cases and there are no clinically approved vaccines or effective drugs to treat WNV infections in humans. The purpose of this study was to facilitate vaccine and antiviral drug discovery by developing a packaging cell line-restricted WNV infectious replicon particle system. We constructed a DNA-based WNV replicon lacking the C-prM-E coding region and replaced it with a GFP coding sequence. To produce WNV replicon particles, cell lines stably-expressing prM-E and C-prM-E were constructed. When the WNV replicon plasmid was co-transfected with a WNV C-expressing plasmid into the prM-E-expressing cell line or directly transfected the C-prM-E expressing cell line, the replicon particle was able to replicate, form green fluorescence foci, and exhibit cytopathic plaques similar to that induced by the wild type virus. The infectious capacity of the replicon particles was restricted to the packaging cell line as the replicons demonstrated only one round of infection in other permissive cells. Thus, this system provides a safe and convenient reporter WNV manipulating tool which can be used to study WNV viral invasion mechanisms, neutralizing antibodies and antiviral efficacy.

## Introduction

West Nile virus (WNV) is a neurotropic flavivirus and the etiologic agent responsible for West Nile encephalitis in humans^1^. Since it was first identified in Uganda in 1937, WNV has been reported in Africa, Asia, Europe, Australia and North America^2^; however, WNV isolate was not reported in China until 2014, despite being endemic in neighbouring countries (e.g., Russia and India)^3^. Lu *et al*. reported the isolation of WNV from mosquitoes in Xinjiang Uyghur Autonomous Region in western China^4^, and Li *et al*. provided evidence of WNV human infections confirmed by an IgM ELISA and the seroconversion of 90% plaque reduction neutralization tests of paired serum samples obtained from persons with febrile illness and viral encephalitis in 2004^5^. Although variety of birds and mammals are susceptible to WNV infection, typically only infected humans and horses exhibit serious symptoms, such as disorientation, coma, paralysis, and potentially death^6^. WNV is transmitted to humans through the bite of infected mosquitoes that acquire the virus after feeding on vertebrate amplifying hosts (i.e., birds).

The WNV genomic RNA contains a single open reading frame (ORF) encoding a long polyprotein, 5′-C-prM(M)-E-NSI-NS2A-NS2B-NS3-NS4A-NS4B-NS5-3′. The structural proteins C (capsid; the C precursor is called anchored C), M (membrane; the M precursor is called prM) and E (envelope) are encoded in the 5′ quarter of the genome and the genes for the non-structural proteins are located in the remaining regions^7^.

There is no specific treatment available for WNV infections and vaccination is the only effective means to prevent WNV infection of humans and animals. The majority of the existing approaches for studying viral neutralization measure the inhibition of viral entry as a reduction in the number of plaques formed in the monolayers of suitable cell lines. However, the wild type virus requires a biosafety level three laboratory (BSL-3), which is associated with both an increased risk and cost of studying WNV^8^. In previous studies^9^, researchers have described a reporter replicon particles system for measuring the neutralizing antibody against WNV. This system involves a sub-genomic replicon capable of expressing a reporter gene and co-transfection of WNV structural proteins expressing plasmids. Moreover, similar packaging systems have been developed for several other flaviviruses, such as WNV replicons^10–12^, Kunjin virus (KUNV)^13^, tick-borne encephalitis virus (TBEV)^14, 15^ and dengue virus^16, 17^. Except for report of tetracycline-inducible packaging cell line^18^, there are few reports about stable cell lines simultaneously expressing all three WNV structural proteins for production of replicon particles. Previously reports described selecting cell lines by using of Sindbis virus replicon (10) and Venezuelan equine encephalitis virus (11) replicon encoding WNV C-prM-E proteins to package the WNV RRPs. As described (11), after serial passages they detected a rapid loss of replicon-encoded reporter gene activity. In both of these cell lines, the WNV structural protein genes were not integrated into the cell genome. Here we successfully established a WNV reporter replicon particles packaging system based genome integrated cell lines simultaneously expressing two (prM-E) or all three (C-prM-E) WNV structural proteins. Moreover, the reporter replicon particles are infectious and the replicon RNA packaged into the reporter replicon particles replicates in infected cells; however, since the viral structural proteins are not encoded by the replicon, only a single round of infection is initiated and subsequent viral progeny cannot be produced. Thus, this single-round of infectivity feature of the reporter replicon particles enables safe handling under biosafety level 2 (BSL-2) conditions.

In this study, we developed reporter replicon particles using two different genetic complementation approaches. When the reporter replicon particles infect BWNV-CME cell lines, they can spread and morphologically alter the infected cells by mimicking the wild type virus, in which plaque formation can be readily visualised and enumerated. Therefore, this reporter replicon system has great potential to facilitate novel drug discovery and vaccine development for WNV.

## Results

### DNA-launched WNV reporter replicon

**Figure 1:**
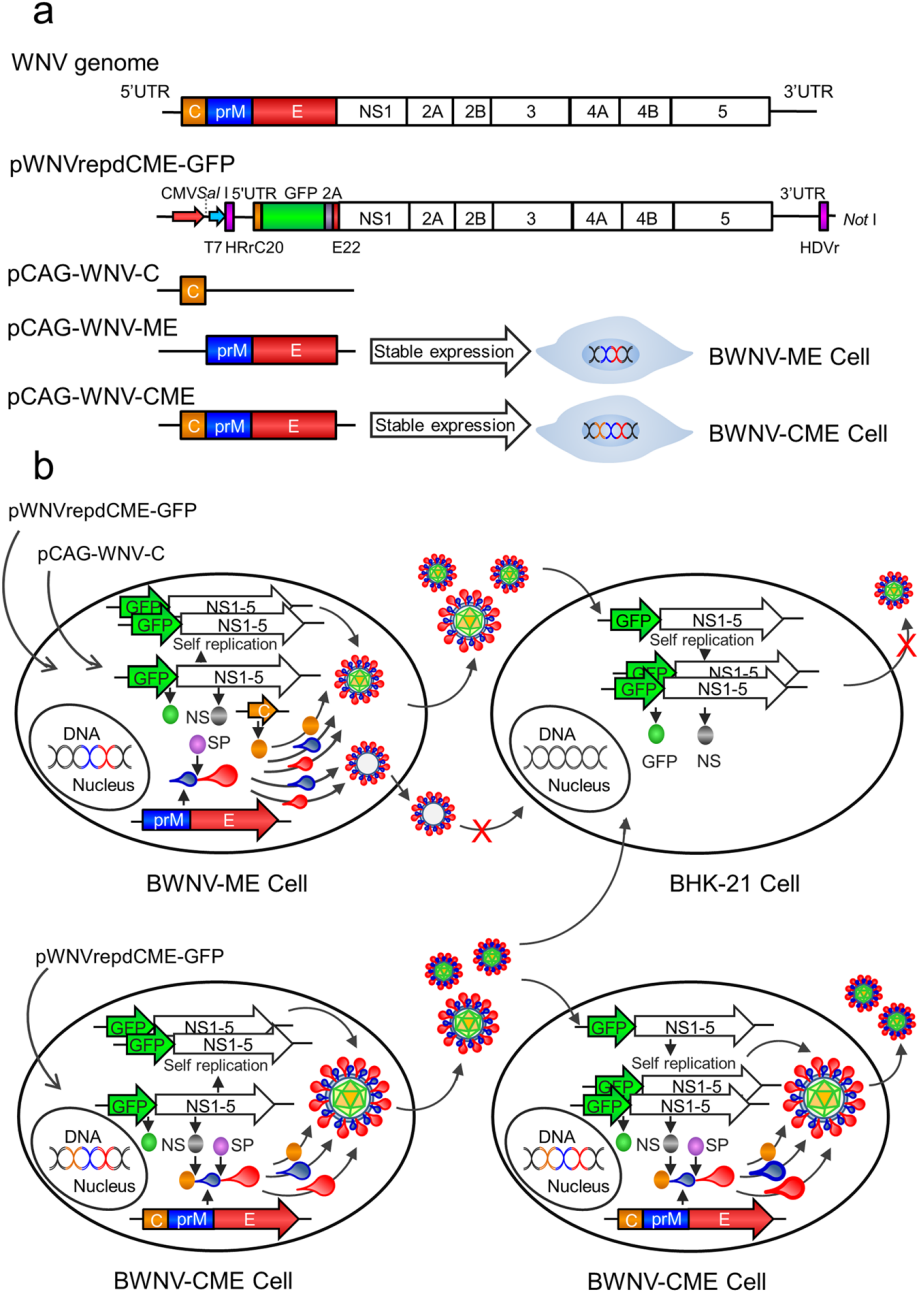
Schematic representation of WNV replicon constructs and packaging of WNV reporter replicon particles (RRPs). (**a**) The DNA based WNV replicon is under control of the CMV promoter. The replicon genome lacks the major coding sequence of the structural protein, C-prM-E, and the corresponding sequence was replaced with a GFP coding sequence following the FMDV 2A coding sequence. The replicon RNA with an authentic 5′ terminus is ensured by placing a hammerhead ribozyme sequence (HRr) before the first 5′ UTR nucleotide. The authentic 3′ terminus is ensured by the addition of a hepatitis delta virus ribozyme sequence (HDVr) after the last 3′ UTR nucleotide. To complement the structural proteins, a capsid protein expressing plasmid was constructed. The BWNV-ME cell line stably expressing the prM-E protein was generated via transfecting BHK-21 cells with the pCAG-WNV-ME plasmid. The BWNV-CME cell line stably expressing the C-prM-E protein was generated by transfecting BHK-21 cells with the pCAG-WNV-CME plasmid. (**b**) WNV RRPs are packaged in two ways. First, the pCAG-WNV-C and replicon plasmids were transfected into BWNV-ME cells. The pCAG-WNV-C plasmid expressed protein C. The cell itself expressed prM and E protein. The replicon plasmid was transcribed by the CMV promoter into the replicon RNA expressing GFP and the non-structural replicase protein. The replicon RNA amplifies itself once again and three structural proteins package the replicon RNA into WNV RRPs which are secreted into the culture medium. The second way is to transfect BWNV-CME cells with only the replicon plasmid. The BWNV-CME cells express the C-prM-E polyprotein which is cleaved into the C, prM and E proteins by replicon RNA-encoded non-structural protease and endogenous cellular signal peptidase (SP). Then three structural proteins package the replicon RNA into RRPs which is secreted into the culture medium. When RRPs infect BHK-21 cells, the replicon RNA expresses GFP and non-structural proteins which amplify more RNA. However, no WNV structural proteins or additional RRPs produced in RRP-infected cells, thereby preventing further spread. When BWNV-CME cells are infected with RRPs, the structural proteins expressed by the cells package the replicon RNA into progeny RRPs and the infection spreads in rounds similar to the wild type virus.

To construct the WNV replicon, the NY99/DQ211652 sequence was used as the reference sequence. The DNA-based WNV replicon was designed as presented in Fig. 1a. The entire length of the replicon genome was divided into four fragments and chemically synthesized. Then, the four fragments were sequentially cloned into a pCI-neo plasmid using an infusion clone assay. *Escherichia coli* JM108 was selected as the strain of host bacteria. The replicon plasmid was verified by sequencing and denoted pWNVrepdCME-GFP. The overall scheme of the pWNVrep dCME-GFP is outlined in Fig. 1a. The replicon plasmid was further verified by transfection of BHK-21 cells and testing GFP expression by fluorescence observation and the level of WNV NS1 protein by a Western blot assay (data not shown).

### Establishment of a BWNV-CME replicon packaging cell line

**Figure 2:**
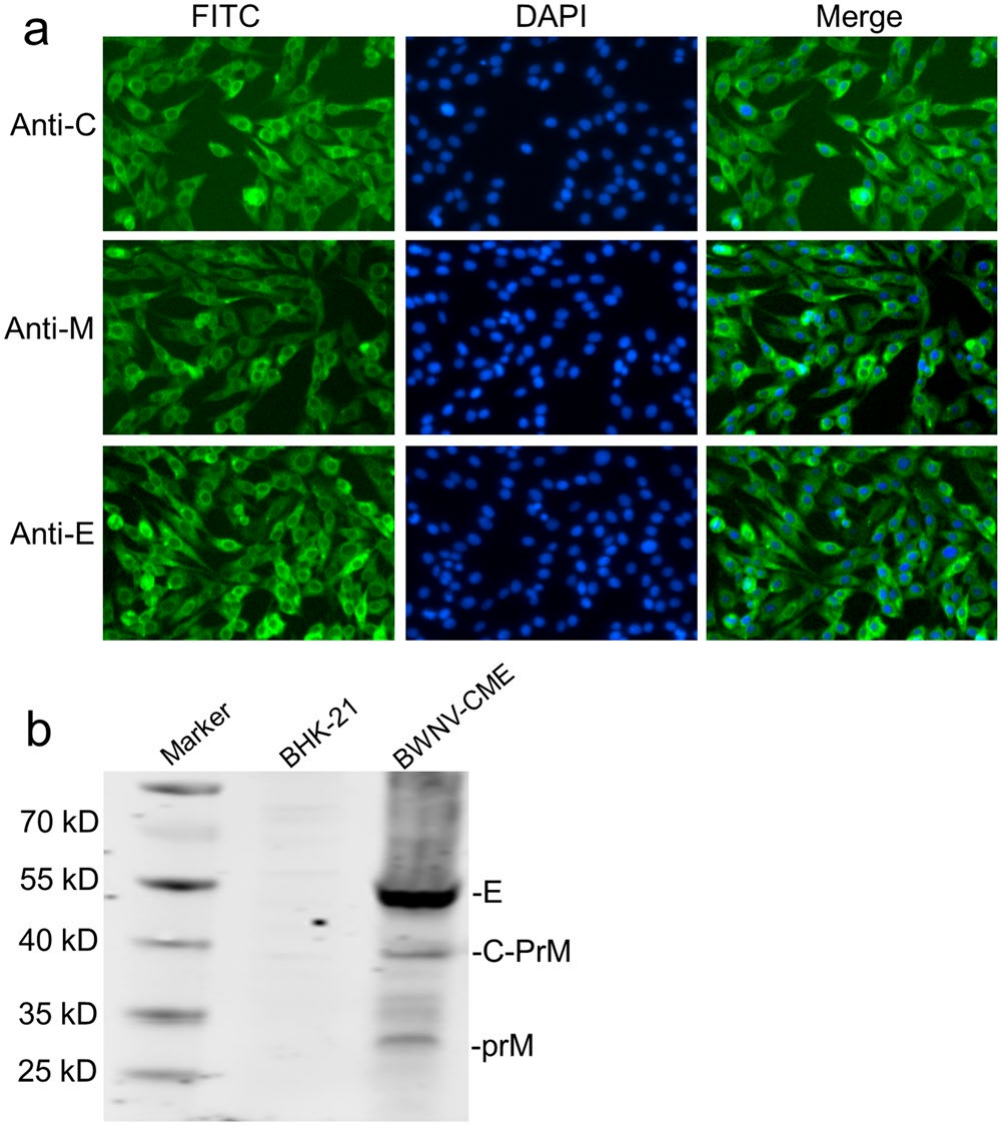
Immunofluorescence and Western blotting analyses of C, prM and E protein expression in BWNV-CME cells. (**a**) Immunofluorescence analysis of BWNV-CME cells with monoclonal antibodies against the C, prM and E proteins. (**b**) BWNV-CME cell lysates were analysed by Western blotting with MAbs against C, prM and E. The cell lysates of BHK-21 cells were used as a negative control. The relative locations of the WNV E, C-PrM and PrM proteins are indicated on the right. Full-length blots are presented in Supplementary Figure 1.

We engineered BHK-21 cells stably expressing WNV C-prM-E proteins by transfecting cells with the pCAG-WNV-CME plasmid (Fig. 1). Following transfection, selection with G418, and after two more rounds of dilution clone, the stable cell line was established and termed, BWNV-CME. The BWNV-CME cells were further identified by an indirect immunofluorescence assay (IFA) and Western blot (WB) with monoclonal antibodies against WNV C, prM and E proteins, respectively. The IFA revealed that all three WNV structural proteins were expressed in the BWNV-CME cells (Fig. 2a). The BWNV-CME cells were also subjected to a WB analysis. As shown in Fig. 2b, the 53 kDa, 38 kDa and 26 kDa bands predicting the size of E, C-prM and prM, respectively were detected in the WNV-CME cells but not in the mock BHK-21 cells, indicating that all the structural proteins were expressed. Taken together, these findings demonstrated that the BWNV-CME cell line was generated and could stably express the WNV C, prM and E proteins.

### Packaged WNV reporter replicon particles replicate and spread within BWNV-CME packaging cells

**Figure 3:**
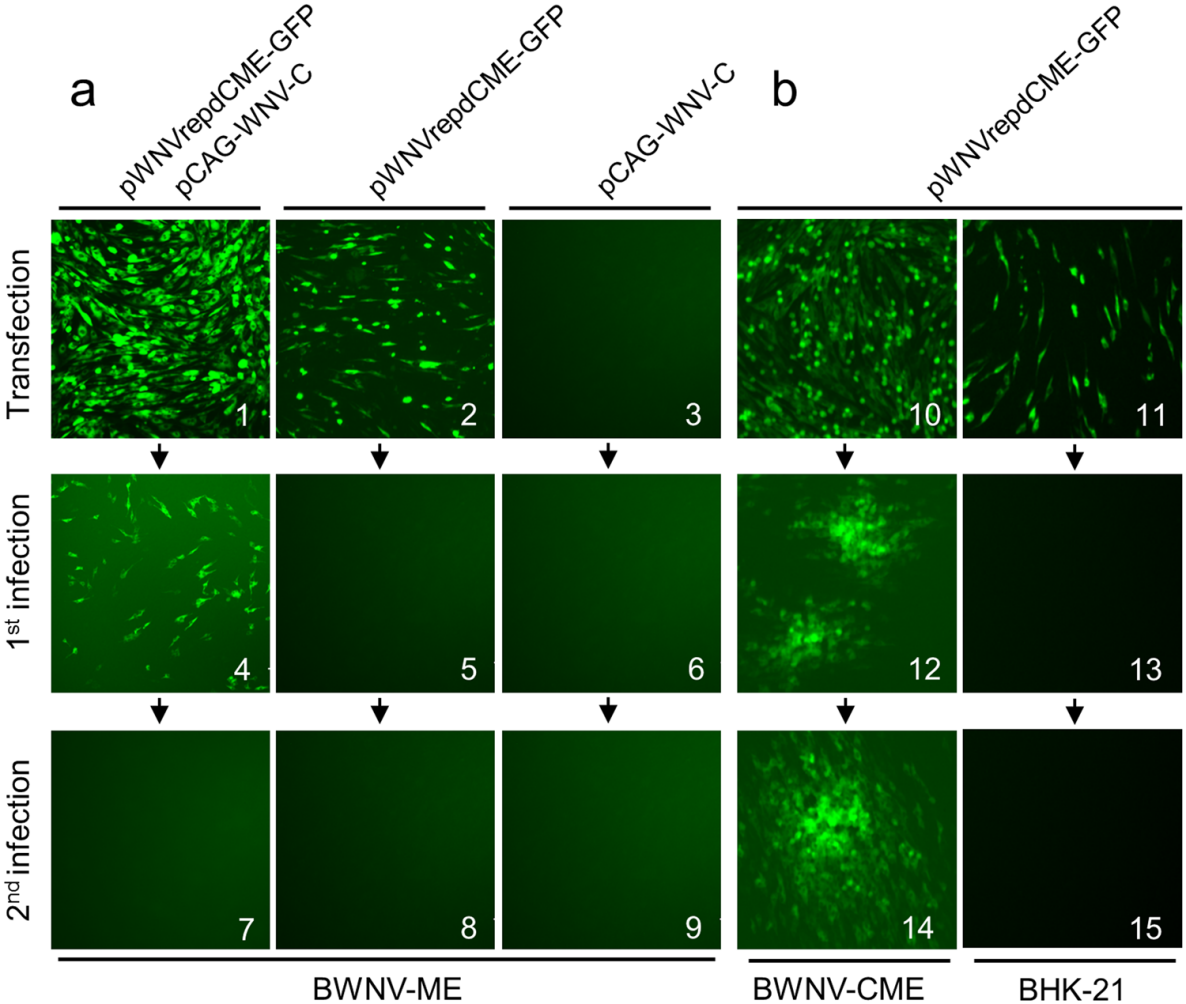
Production of RRPs from the WNV replicon plasmid by transfecting (**a**) BWNV-ME and (**b**) BWNV-CME cell lines. Green florescence was visualized when the replicon plasmid pWNVrepdCME-GFP was transfected into BWNV-ME cells (2), BHK-21 cells (11), BWNV-CME cells (10) or BWNV-ME cells together with the pCAG-WNV-C plasmid (1). The transfected cell supernatants were passaged onto fresh cell cultures as indicated by the arrows, and green florescence was observed only in the cells labelled 4 and 12. Three days post-inoculation, the supernatants of the cells infected first were used to inoculate the cells as indicated by the arrows for a second infection. Green florescence was analysed 72 h post-infection.

The WNV reporter replicon particles (RRPs) here named ΔWNV-GFP were packaged using two strategies (Fig. 1b). The infectivity characteristics of the packaged ΔWNV-GFP were surveyed with BHK-21, BWNV-ME and BWNV-CME cells. Supernatants from the transfected cells were harvested three days post-transfection for virus collection. The ΔWNV-GFP infected BHK-21 cells expressed the GFP reporter gene which permitted only one round of infection. The ΔWNV-GFP-infected BWNV-ME cell supernatants were not able to infect the BWNV-ME cells in the second round (Fig. 3a). However, ΔWNV-GFP could infect BWNV-CME cells and produce additional RRP progeny (Fig. 3b). As the infection time increased, the number of GFP-expressing cells grew and formed fluorescence foci. These results demonstrated that the ΔWNV-GFP can indeed only replicate once in normal cells, but exhibit replication characteristics similar to that of the wild type virus.

### Infectious properties of ΔWNV-GFP

**Figure 4:**
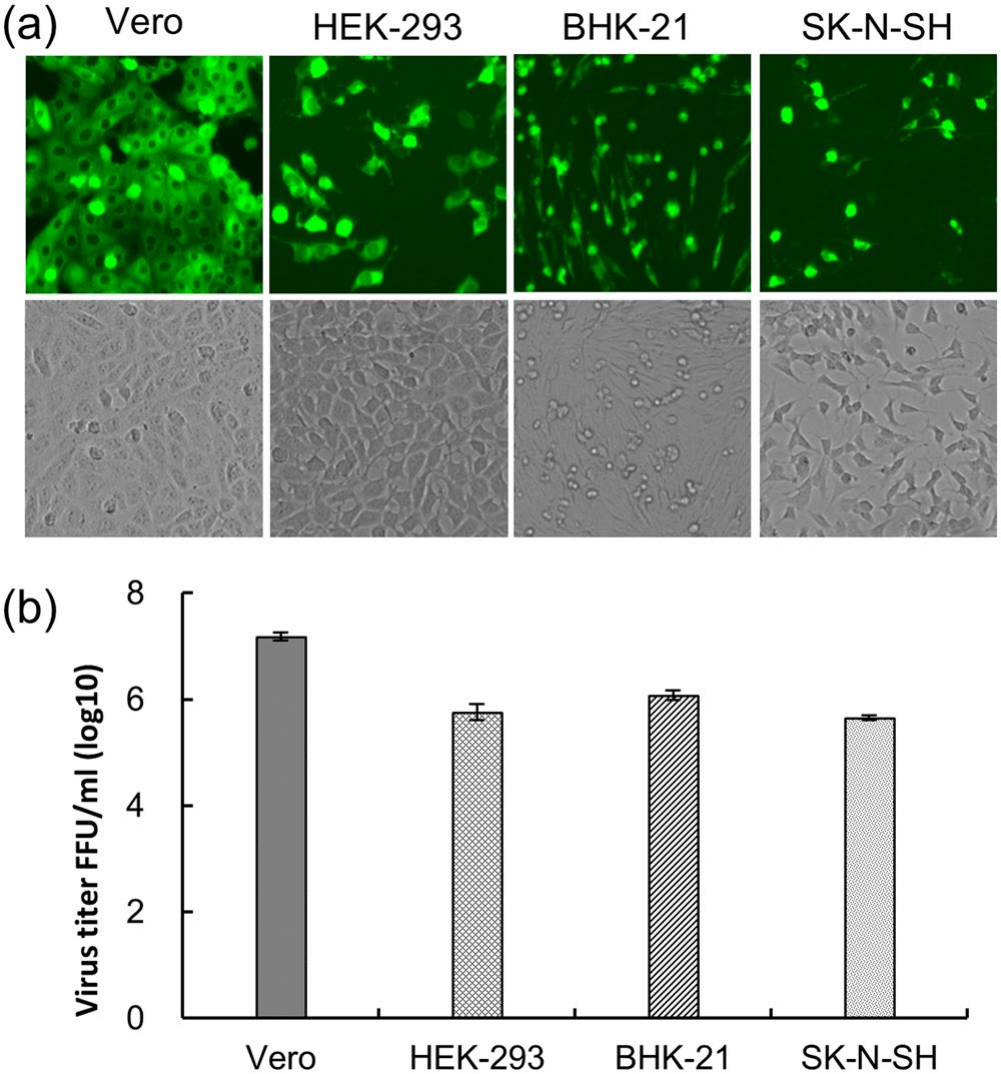
Infectious properties of WNV RRPs in Vero, HEK-293, BHK-21 and SK-N-SH cells. (**a**) Expression of the GFP reporter gene in each cell line after the infection of equal amounts of RRPs. (**b**) Monolayers of each cell line in a six-well plate containing 10^5^ to 10^6^ cells/well were infected in parallel with 10-fold serial dilutions of WNV RRPs. Cells positive for GFP protein were counted at the appropriate dilution and the number of RRPs per 1 mL of supernatant were calculated.

The infectious properties of ΔWNV-GFP were tested on flavivirus susceptible cells, including Vero, HEK-293, BHK-21 and SK-N-SH cells. Equal amounts of ΔWNV-GFP were used to infect the four cell lines. In total, the titres applied to BHK-21 were approximately 10-fold lower than that used for the Vero cells (Fig. 4). The results were fairly consistent with the experimental results reported in the literature^10^. Moreover, the SK-N-SH cells were similar to HEK-293 cells with regards to their susceptibility to WNV infection.

### ΔWNV-GFP replication kinetics

**Figure 5:**
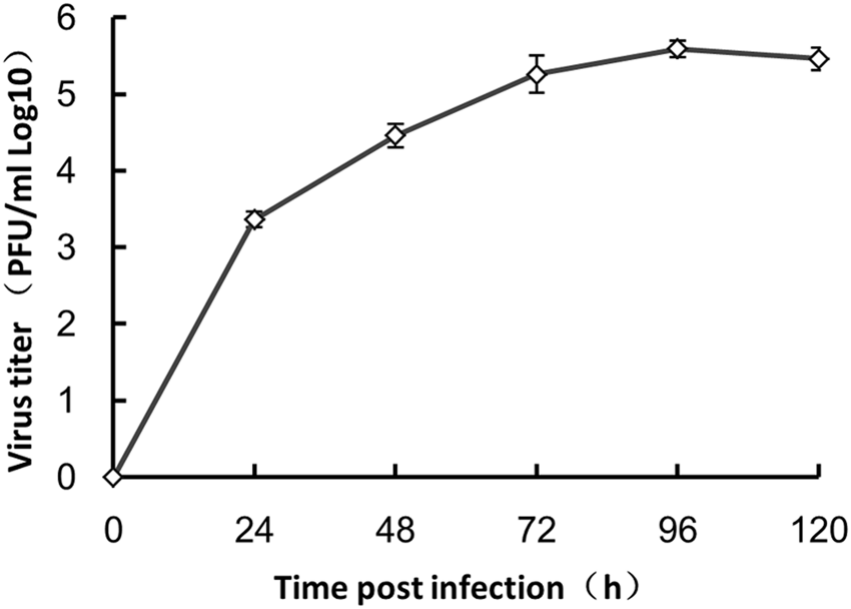
Accumulation of RRPs in the culture fluid of RRPs infected BWNV-CEM cells analysed by an infectivity assay. The titres are expressed as the total number of RRPs per mL supernatant of infected BWNV-CME cells.

To precisely estimate the ΔWNV-GFP titre produced by infected BWNV-CME cells and to analyse their secretion kinetics over time, we collected the culture fluid from infected BWNV-CME cells at daily intervals and analysed it using infectivity assays. The ΔWNV-GFP infectious titre reached 10^6^ on the third day post-infection and virus secretion was maintained at a similar level until the fifth day post-transfection (Fig. 5). These results demonstrate that the infected BWNV-CME cells produced and continually secreted infectious virus for an extended period.

### Neutralizing antibody testing

**Figure 6:**
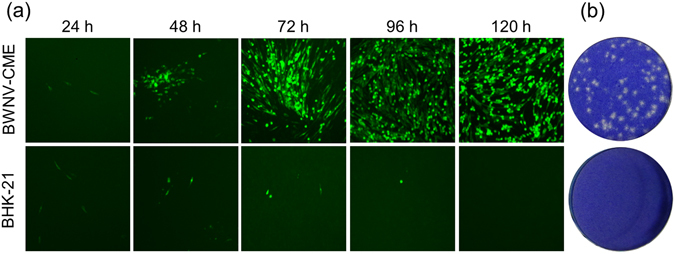
Propagation characteristics of RRPs in BWNV-CME and BHK-21 cells. (**a**) The spread of green fluorescence in BWNV-CME cells infected with RRPs. In RRP-infected BHK-21 cells, the green fluorescence expressing cells did not increase from 24 h post-infection. The BWNV-CME and BHK-21 cells were seeded into 24-well plates and inoculated with RRPs for the indicated time periods. For detection of plaques, the infected cells were overlaid with Eagle’s medium containing 1.5% carboxymethyl cellulose and 1% foetal calf serum. The morphological changes of one plaque were observed with a fluorescent microscope. (**b**) WNV plaque formation in WNV-CME cells. Six days post-infection, the plates were stained with 0.1% crystal violet solution for 15 min at room temperature. The stain was discarded and the cells were rinsed with tap water.

**Figure 7:**
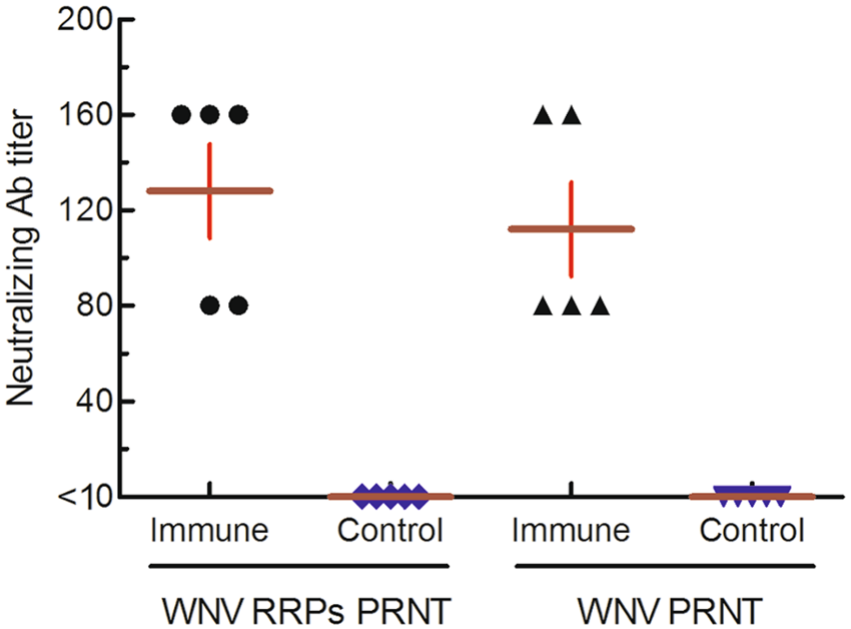
Mouse sera neutralizing antibody detected by PRNT with ΔWNV-GFP and the WNV native virus. The dark red horizontal lines represent the average neutralizing antibody titre.

The sample set used in the PRNT evaluation consisted of 30 serum specimens performed in duplicate with assays from mice, geese, and horses immunized with WNV prM-E (prM-E, BWNV-ME cells expressed virus-like particles, unpublished data), and several flavivirus-negative controls. The mouse sera had been characterized previously by ELISA and PRNT detection with WNV (unpublished data). Since the BWNV-CME cells infected with ΔWNV-GFP could package more RRP progenies and result in a greater number of infected cells, the GFP fluorescent-positive cells accumulated and formed fluorescent foci (Fig. 6a). A five-day incubation of ΔWNV-GFP with WNV-CME cells resulted in the formation of highly discrete plaques (Fig. 6b). The plaques were well-defined and easily discernible; their small size was indicative of a slow growing virus. Thus, the method of virus titration described here enables the determination of the correct virus dilution for use in PRNT. For mouse sera, the results obtained by ΔWNV-GFP PRNT and WNV PRNT demonstrated good concordance (Fig. 7). All goose and horse sera positive in the ELISA were also positive by PRNT (Table 1). These results indicate that the ΔWNV-GFP/BWNV-CME cell system could be used for testing neutralizing antibodies.

**Table 1:** Neutralizing antibodies derived from goose and horse sera.

No.	PRNT titer^a^		ELISA	
	Immune	Control	Immune	Control
Goose 1	320	<10	+	−
Goose 2	160	<10	+	−
Goose 3	80	<10	+	−
Goose 4	320	<10	+	−
Goose 5	160	<10	+	−
Horse 1	160	<10	+	−
Horse 2	160	<10	+	−

^a^The PRNT titer was expressed as the maximum serum dilution yielding a 50% plaque reduction.

### WNV inhibitors blocked RRP replication in BWNV-CME cells

**Figure 8:**
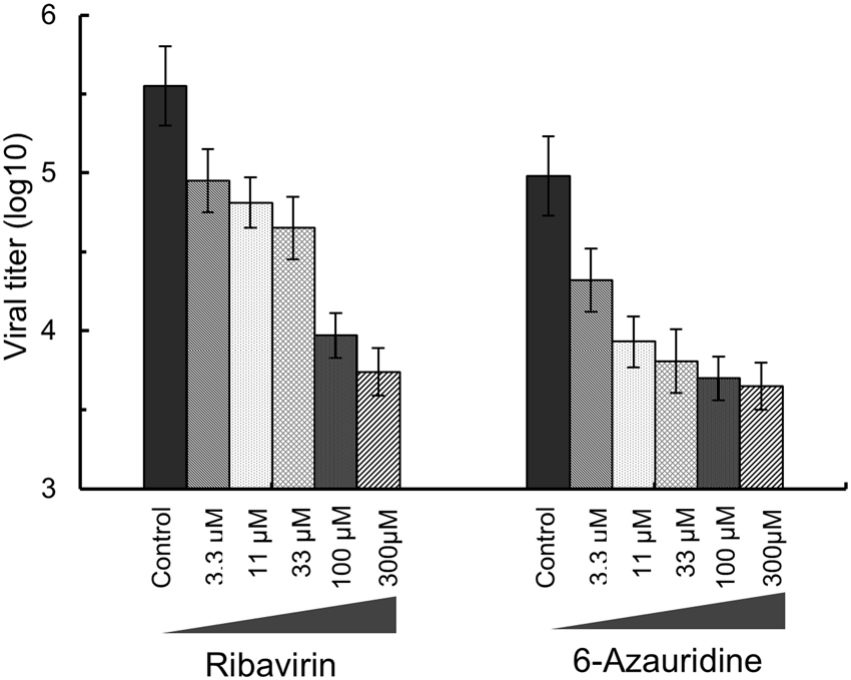
The reporter replicon particles can be used for antiviral drug discovery. BWNV-CME cells were infected with the indicated viruses at an MOI of 0.1; the infected cells were immediately treated with ribavirin and 6-Azauridine. For WNV, the culture media were collected at 30 h and viral titres were measured using plaque assays.

For the antiviral assay, BWNV-CME cells were infected with ΔWNV-GFP at a multiplicity of 0.1 PFU per cell in the presence of 0, 3.7, 11, 33, 100 and 300 μM of compound. The culture supernatants were harvested at 32 h and the viral titres were determined using plaque assays as previously described. The compound inhibited the viral titre in a dose-responsive manner (Fig. 8). The results showed that ribavirin and 6-Azauridine exhibited an inhibitory effect on virus production, suggesting that the ΔWNV-GFP reporter system can be applied to screen for potential WNV inhibitors.

## Discussion

In this study, we constructed a WNV cDNA clone lacking structural genes under the control of a cytomegalovirus (CMV) promoter. In addition, the structural protein, C-prM-E, was replaced with GFP. Compared to most replicon RNAs which are produced by *in vitro* RNA synthesis using constructs in which the subgenomic RNA is placed downstream of a bacteriophage promoter (e.g., T7), our replicon allows for the production of replication-defective replicon particles following the transfection of cells with plasmid DNA using standard methods. Moreover, replicons are also designed to encode GFP genes that are visible following infection with ΔWNV-GFP and were shown to be a useful marker for the analysis of infection with the virus. We also employed two different methodologies to introduce the vector cassette into the packaging cell line. In one approach, we used pCAG-WNV-C and pWNVrepdCME-GFP generated by transient transfection to transduce the BWNV-ME packaging cell lines; this methodology is highly efficient and titres can reach as high as 5 × 10^6^ FFU/mL. While ΔWNV-GFP does require the use of expensive transfection reagents and the number is often limited, we also described the production of ΔWNV-GFP via the complementation of replicons with BWNV-CME cells. The advantage of this second approach lies in its simplicity, and that it allows for the production of a large number of flavivirus variants by simply infecting BWNV-CME cells with supernatant containing ΔWNV-GFP.

WNV belongs to the Japanese encephalitis virus serogroup. Viruses in this serogroup may cause cross-reactions and affect the specificity of various serological assays used for viral testing. PRNT is the most specific serological test used for the identification of flaviviruses^19, 20^ and is considered to be the gold standard protocol for the serodiagnosis of flavivirus infection. However, although the traditional PRNT method must be handled under BSL-3 conditions, experiments that use our systems can be conducted under BSL-2 conditions. To verify whether ΔWNV-GFP can act as a substitute for WNV in neutralizing antibody detection and antiviral drug screening, we investigated the biological properties of ΔWNV-GFP (e.g., infectious properties, plaque morphology and growth kinetics). Our findings indicate that ΔWNV-GFP exhibited biological properties indistinguishable from the wild type virus. Our replicon particles system is associated with several advantages: (1) the virus readily forms plaques in BWNV-CME cells and can be used to investigate the neutralization of WNV; (2) the standard PRNT approach involves the use of live infectious virus, which must be handled by a skilled investigator in an appropriate biocontainment facility, whereas our replicon particles system does not require BSL3 containment. In addition, from testing the infectious properties of WNV, the application of ΔWNV-GFP also has several desirable features that complement PRNT. The level of ΔWNV-GFP infection in cells is measured directly as a function of reporter gene activity, allowing for the study of virus entry. Moreover, the inhibition of viral entry can also be tested using a variety of cell types, including those that do not support the formation of plaques. Therefore, the establishment of stable BWNV-CME cell lines facilitates the usage of this WNV replicon system, rendering it a more suitable tool for studying the mechanisms of WNV invasion, vaccine development and antiviral research.

## Materials and Methods

### Cells, plasmids, and antibodies

Baby hamster kidney (BHK-21) (CCL-10; ATCC), African green monkey kidney (Vero), HEK-293 and human neuroblastoma-derived SK-N-SH cells were cultured in Dulbecco’s modified Eagle’s medium (DMEM; Gibco, Invitrogen, Carlsbad, CA) supplemented with 10% foetal bovine serum (FBS; Gibco, Grand Island, NY) and 100 U/mL penicillin and maintained in 5% CO_2_ at 37 °C. The BWNV-ME cell line which stably expresses the WNV prM-E protein was previously generated (unpublished data) by the transfection of BHK-21 cells with a prM-E-expressing plasmid. Monoclonal antibodies (MAbs) against the WNV C protein was purchased from Gene Tex (Texas, USA). MAbs against the WNV prM and E proteins were generated in our laboratory^21^.

### DNA-launched subgenomic replicon

A cDNA clone of WNV strain NY99 (GenBank accession no. DQ211652) lacking the *C-prM-E* gene was constructed by chemical synthesis. Briefly, the full-length genome sequence was divided into four fragments and artificially synthesized by a DNA synthesis company (BOSHI, Harbin, China) and cloned into the eukaryotic expression vector pCI-neo at the *Xho* I and *Xba* I sites. This was performed to ensure the production of flavivirus RNA with an authentic start and terminus following transcription by a cellular RNA polymerase in the nucleus of the transfected cells. The replicon encodes an HRr ribozyme at the 5′ end and a hepatitis delta virus (HDV) ribozyme followed by a SV40 polyadenylation signal at the 3′ end. We also introduced GFP genes in place of the structural genes to facilicate to visualise replication of the replicon virus at the 5′ end. In addition, the 2A autoprotease of foot and mouth disease virus (FMDV) was cloned downstream of the insertion, which can liberate the GFP gene from the flavivirus polyprotein during translation (Fig. 1).

### Establishment of a stable cell line continuously expressing the WNV C-prM-E protein

First, genetic codon-optimized WNV cDNA encoding the viral structural protein C-prM-E was synthesized and digested with *Sac* I and *Xho* I. Then, the target DNA fragment was inserted into the *Sac* I and *Xho* I sites of the expression vector pCAGneo^22^ to generate pCAG-WNV-CME. The pCAGneo plasmid contains the neomycin resistance gene, which confers resistance to G418 (EMD Chemicals Inc., San Diego, CA, USA). A monolayer or about 90% confluent BHK-21 cells were transfected with the pCAG -WNV-CME plasmid using FuGENE HD transfection reagent (Roche Diagnostic GmbH, Mannheim, Germany). Two days later, the transfected cells were digested and cloned by limited dilution in 96-well plates and growth in medium containing G418. The cloned cells were selected using an indirect immunofluorescence assay (IFA) with a WNV E protein-specific monoclonal antibody. The amount of E antigen produced by the cloned cells was examined and compared by Western blotting. One clone (designated BWNV-CME) that exhibited more efficient expression of the E antigen was selected and maintained in G418-supplemented medium for further characterisation and antigen production.

### Immunofluorescence assay and Western blotting analysis

The level of BWNV-CME antigen expression in infected cells or cell lysates were analysed by IFA and Western blot analysis in accordance with the procedure described previously^22^.

### DNA transfection and production of reporter replicon particles

To trans-complement the C protein, a C protein-expressing plasmid, pCAG-WNV-C, was constructed by inserting the codon-optimized WNV C protein encoding sequence into the *Sac* I and *Xho* I sites of the expression vector, pCAGneo^22^. At 80–90% confluency, BWNV-CME cells were transfected with plasmid pWNVrepdCME-GFP and BWNV-ME cells were transfected with pCAG-WNV-C and pWNVrepdCME-GFP using the FuGENE 6 transfection reagent (Roche Diagnostic GmbH, Mannheim, Germany) in accordance with the protocol recommended by the manufacturer. The transfected cells were maintained in DMEM with 5% FBS for three to four days in a humidified 5% CO_2_ chamber. The culture media from the transfected cells was collected and stored at −80 °C until further use. The replicon particles packaged using the two strategies were denoted as ΔWNV-GFP.

### Single round infectivity and restricted continuous replication of ΔWNV-GFP

The infectivity characteristics of the packaged ΔWNV-GFP reporter particles were surveyed with BHK-21, BWNV-ME and BWNV-CME cells. Briefly, the cellular monolayer was inoculated with the supernatant of the transfected cells containing the packaged ΔWNV-GFP reporter particles. GFP fluorescence was observed at various time points post-infection. The supernatants from the infected cells were harvested 72 h post-infection and used to infect each cell line for a second and third round. The expression of GFP was examined to determine whether infectious replicon particle progeny was produced following infection with ΔWNV-GFP.

### Preparation of WNV reporter replicon particles and titration

To conveniently prepare a sufficient amount of ΔWNV-GFP, BWNV-CME cells were infected with the supernatants harvested following transfection at an MOI of 0.1 in 1 × MEM plus 5% FBS. ΔWNV-GFP in the supernatants was collected three to four days post-infection and the supernatants were filtered using a 0.45 μm filter system. The viral titres were determined using a fluorescence unit counting method following infection of BHK-21 or other permissive cells. When infecting BWNV-CME cells, the virus could be tittered using a plaque assay. BWNV-CME cells grown in 24-well plates were infected with the indicated virus in a 10-fold serially diluted manner. After the cells had been infected for 6 h at 37 °C, they were overlaid with medium (1.5% carboxymethyl cellulose and 1% foetal calf serum in Eagle’s medium). After a six-day incubation, the plate was stained in a 0.1% crystal violet solution for 15 min at room temperature. Then the stain solution was discarded, the cells were rinsed with distilled water and the plaques were counted.

### Infectious properties of RRPs

The efficiency of infection *in vitro* with the same preparation of WNV differs among different cell lines. To determine whether the WNV replication-defective virus demonstrates the same cell line-dependent specific infectivity, Vero, BHK-21, HEK-293 and SK-N-SH cells were infected in parallel with serial 10-fold dilutions of WNV. The cells that were positive for GFP protein expression were counted at the appropriate dilution, and the number of virus per 1 mL of the supernatant was calculated.

### Replication kinetics study

To precisely estimate the number of reporter replicon particles produced by an infection and analyse the replicon secretion kinetics over time, we collected the culture fluid from infected cells at daily intervals and analysed it using BWNV-CME cells by performing plaque assays.

### Testing WNV neutralizing antibodies with RRPS in BWNV-CME cells

The presence of WNV neutralizing antibodies were tested using a plaque reduction assay. The test procedure was performed as follows: (1) the sera were inactivated for 30 mins in a water bath at 56 °C; (2) serial dilutions of the sera were made in cell culture medium from 1/10 to 1/320 using a 24-well flat-bottomed microplate; (3) the stock virus was diluted to make 100 plaque-forming units (PFU)/0.2 mL in cell culture medium; (4) one volume of each diluted serum sample was mixed with an equal volume of the diluted virus. A virus control with culture medium, negative serum control and positive serum control were included in each plate; (5) the plate was incubated for 90 min at 37 °C; (6) then 100 µL of the virus and serum mixture was added to the wells containing a WNV-CME cell monolayer formed on a 24-well culture plate; (7) the plates were incubated in a CO_2_ atmosphere for 90 min at 37 °C; (8) the inoculum was removed and 1 mL of overlay medium (1.5% carboxymethyl cellulose and 1% foetal calf serum in Eagle’s medium) was added; (9) the plates were incubated in a CO_2_ atmosphere for five to six days at 37 °C; and (10) after removing the culture fluid, the plate was stained in a 0.1% crystal violet solution for 15 min at room temperature. The stain was discarded and the cells were rinsed with tap water. The cells were air-dried and the plaques were counted. The dilution of serum that was required to reduce the number of plaques by 50% of the control without serum was estimated. The neutralizing antibodies of mouse sera were tested with live WNV in BHK-21 cells according to previous reports^23^.

### Antiviral assays

To survey whether the RRP and BWNV-CME cell system could be used to screen inhibitors of WNV, two reported WNV inhibitors, ribavirin and 6-Azauridine^24^, were tested for their inhibiting ability against RRPs in BWMV-CME cells. Viral titre reduction assays were performed to examine the antiviral activity of ribavirin and 6-Azauridine. Approximately 9 × 10^5^ WNV-CME cells/well were seeded into a 24-well plate. The cells were infected with individual virus (MOI of 0.1) and treated immediately with the compound at the indicated concentrations (0, 3.3, 11, 33, 100 and 300 μM, respectively). The compounds were dissolved in DMSO and added to the cells at various concentrations in the medium with a final DMSO concentration of 1%. Cells not treated with the compounds were treated with 1% DMSO as a negative control. After 32 h, the supernatant was collected and the virus titre was measured.

## Electronic supplementary material

Supplementary Information
