# Supplementary material for: West Nile virus infectious replicon particles generated using a packaging-restricted cell line is a safe reporter system
Source: Sci Rep. 2017 Jun 12;7:3286. doi: 10.1038/s41598-017-03670-4 (PMC5468312; doi:10.1038/s41598-017-03670-4)
Supplement: Supplementary file 1 — Supplementary Information [file 41598_2017_3670_MOESM1_ESM.doc]

**West Nile virus infectious replicon particles generated using a packaging-restricted cell line is a safe reporter system**

Wei Li^1^, Le Ma^1^, Li-Ping Guo^1^, Xiao-Lei Wang^1^, Jing-Wei Zhang^1^, Zhi-Gao Bu^1*^, Rong-Hong Hua^1*^

^1^ State Key Laboratory of Veterinary Biotechnology, Harbin Veterinary Research Institute of Chinese Academy of Agricultural Sciences, Harbin, 150001, China.

WL: 907920690@qq.com

LM: 15369300569@189.com

LPG: guoliping_0646@126.com

WLW: wxlsdau@126.com

JWZ: 891963994@qq.com

ZGB: buzhigao@caas.cn

RHH: huaronghong@163.com

^*^ Correspondence and requests for materials should be addressed to RH. Hua. (email: [huaronghong@163.com](mailto:huaronghong@163.com)) or ZG. Bu. (email: [buzhigao@caas.cn](mailto:buzhigao@caas.cn) )State Key Laboratory of Veterinary Biotechnology, Harbin Veterinary Research Institute, Chinese Academy of Agricultural Sciences. No.678 Haping Road, Harbin 150069, P.R. China


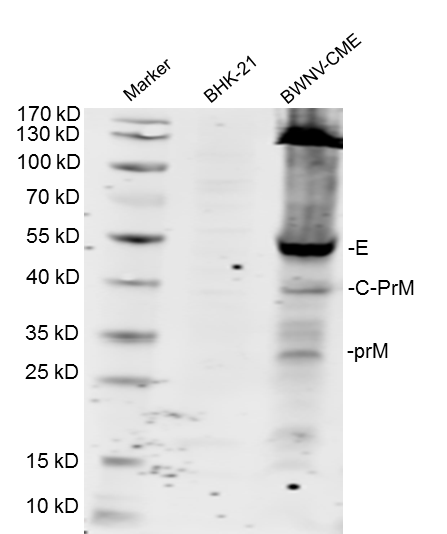


**Supplementary Figure 1.** BWNV-CME cell lysates were analysed by Western blotting with MAbs against C, prM and E. The cell lysates of BHK-21 cells were used as a negative control. The relative locations of the WNV E, C-PrM and PrM proteins are indicated on the right.
